# Scale-Up Evaluation of a Composite Tumor Marker Assay for the Early Detection of Renal Cell Carcinoma

**DOI:** 10.3390/diagnostics10100750

**Published:** 2020-09-25

**Authors:** Dong Su Kim, Won Sik Ham, Won Sik Jang, Kang Su Cho, Young Deuk Choi, Suki Kang, Bora Kim, Kook Jin Kim, Eun Ji Lim, Sun Young Rha, Ja Hyeon Ku, Cheol Kwak, Hyeon Hoe Kim, Chang Wook Jeong, Nam Hoon Cho

**Affiliations:** 1 Genomine Research Division, Genomine, Inc., Pohang Technopark, Pohang, Kyungbuk 790-834, Korea; dskimi@genomine.com (D.S.K.); pupybr@genomine.com (B.K.); gjkim@genomine.com (K.J.K.); limej@genomine.com (E.J.L.); 2 Department of Urology, Yonsei University College of Medicine, 50-1 Yonsei-ro, Seodaemun-gu, Seoul 03722, Korea; uroham@yuhs.ac (W.S.H.); sindakjang@yuhs.ac (W.S.J.); kscho99@yuhs.ac (K.S.C.); youngd74@yuhs.ac (Y.D.C.); 3 Department of Pathology, Yonsei University College of Medicine, 50-1 Yonsei-ro, Seodaemun-gu, Seoul 03722, Korea; hee74@yuhs.ac; 4 Department of Internal Medicine, Yonsei University College of Medicine, 50-1 Yonsei-ro, Seodaemun-gu, Seoul 03722, Korea; RHA7655@yuhs.ac; 5 Department of Urology, Seoul National University Hospital, Seoul National University College of Medicine, Seoul 03080, Korea; kuuro70@snu.ac.kr (J.H.K.); mdrafael@snu.ac.kr (C.K.); hhkim@snu.ac.kr (H.H.K.)

**Keywords:** renal cell carcinoma, biomarker, multiplexed immunoassay, diagnosis

## Abstract

The early detection of renal cell carcinoma (RCC) using tumor markers remains an attractive prospect for the potential to downstage the disease. To validate the scale-up clinical performance of potential tumor markers for RCC (as a single marker and as a composite tumor marker composed of nicotinamide N-methyltransferase (NNMT), L-Plastin (LCP1), and non-metastatic cells 1 protein (NM23A)), the scale-up assay was performed. Patients with RCC from multiple domestic institutes were included in the clinical evaluation for reassessment and improvement of the established triple markers of our product. For the diagnostic performance of the composite markers, the best-split cutoff points of each marker (147 pg/mL for NNMT, 1780 pg/mL for LCP1, and 520 pg/mL for NM23A) were installed. Serum levels of NNMT, LCP1, and NM23A were greatly increased in subjects with RCC (*p* < 0.0001). In 1042 blind sample tests with control individuals (n = 500) and patients with RCC (n = 542), the diagnostic sensitivity and specificity of the composite three-marker assay were 0.871 and 0.894, respectively, and the resulting AUC (Area under Curve) of ROC (Receiver Operating Characteristic) was 0.917. As a single marker, the diagnostic accuracies of NNMT, LCP1, and NM23A, as estimated by ROC, were 0.833, 0.844, and 0.601, respectively. The composite three-marker assay with NNMT, LCP1, and NM23A is a more improved novel serum marker assay for the early detection of RCC in cases of renal mass or unknown condition. The NNMT, LCP1, and NM23A triple marker assay could be a powerful diagnostic tumor marker assay to screen the early stage of RCC.

## 1. Introduction

Renal cell carcinoma (RCC) is the third most common urological malignancy, representing approximately 2–3% of all malignancies worldwide, and it is notorious for having a dismal prognosis [1]. The three major subtypes of RCC, clear cell, papillary, and chromophobe, are classified based on histologic and cytogenetic features and have been well correlated with biologic behavior [2,3]. The zone of RCC is quite variable in prognosis, ranging from indolent behavior in chromophobe RCC to aggressive behavior in the clear cell subtype of RCC with the loss of chromosome 3p [2,3]. Due to the lack of curative therapy and high metastasis rate (up to approximately 30% overall and 15–25% at presentation), RCC is one of the most refractory malignancies and is very refractory to conventional chemotherapy [1,2,3]. Renal cancer is notorious for contributing to a greater average number of years of life lost than colorectal or prostate cancer [4].

The established risk factors for RCC include alcohol consumption and occupational exposure to trichloroethylene (TCE), as well as three well-known factors—cigarette smoking, obesity, and hypertension [5]. The incidence of RCC is perpetually increasing up to 50% [6]; it is more prevalent in developed countries, which is probably associated with an increased detection of renal masses due to imaging modalities. In general, the diagnostic procedure of renal masses is a tedious, laborious, and incidental process, with initial screening by corporal imaging methods and subsequent histological analysis. Furthermore, asymptomatic RCC could be a leading cause of missing detection of renal mass. Despite the importance of early diagnosis and screening for renal masses, little success in screening for RCC has been achieved over the last decade [7,8]. We previously reported the identification and verification of triple markers for the early detection of RCC and suggested their potential as promising biomarkers [9]. Nicotinamide N-methyltransferase (NNMT), L-plastin (LCP1), and nonmetastatic cells 1 protein (NM23A) were explored to evaluate the diagnostic potential of these serum/plasma markers. A feasible multiplex (three-plex) assay for composite markers was also developed to evaluate the analytical and clinical performance [10]. In this study, an expanded clinical study was performed to evaluate the clinical performance of this three marker assay with more scale-up multi-center samples.

## 2. Materials and Methods

### 2.1. Clinical Samples

A total of nine institutes participated in this study by providing healthy or patient specimen collections. Serum samples were obtained from these institutes after obtaining written informed consent and under reviewed the Institutional Review Board of the Severance Hospital (IRB no. 4-2016-1086, 2 February 2017). Participant demographics are summarized in Table 1. Patient serum was collected the day before surgery from 542 subjects within one to six weeks after diagnosis [11]. Patients were diagnosed with RCC by imaging modalities and confirmed by histopathologic analysis. Control serum (n = 500) was obtained from healthy individuals. Serum from healthy individuals were obtained from Kyungpook National University Hospital (n = 150), Ajou University Hospital (n = 200), Jeju National University Hospital (n = 50), and the Korea Institute of Radiological and Medical Sciences (KIRAMS) Radiation Biobank (KRB) (n = 100). Patient serum (n = 542) was obtained from Chonbuk National University Hospital (n = 7), Gyeongsang National University Hospital (n = 11), Pusan National University Hospital (n = 18), Chonnam National University Hwasun Hospital (n = 77), and Seoul National University Hospital (n = 429).

### 2.2. Sample Preparation

Only serum samples were used as the biospecimens for this study. Serum samples were collected under controlled guidelines and by standardized operating procedures reviewed by the National Korea Biobank. In brief, after collection of whole blood in a serum-separating tube (SST) or plain tube, it was kept at 4 °C for up to 1 h before separation. Clots were removed by centrifuging at 1000–2000× *g* for 10 min. The serum was divided into five aliquots and stored at −80 °C.

### 2.3. Recombinant Antigens and Antibodies

Recombinant protein production as antigens or calibrators, antibody generation, and biotin conjugation of detection antibodies was performed as described previously [9,10]. In brief, recombinant proteins were prepared from cDNA for full-length NNMT (NM_006169), LCP1 (NM_002298.4), and NM23A (NM_198175.1).

### 2.4. Antibody Conjugation to Beads

For the microsphere immunoassay, antibody conjugation was performed according to the manufacturer’s instructions. All procedures were done at room temperature. In brief, anti-NNMT IgG, anti-LCP1 IgG, and anti-NM23A IgG were conjugated to bead 63, bead 17, and bead 33 (Luminex Corp., Austin, TX, USA) respectively. Beads washed with deionized water were transferred to phosphate buffer. *N*-hydroxysulfosuccinimide sulfo-NHS (NHS) and 1-ethyl-3(3-dimethylaminopropyl)-carbodiimide hydrochloride (EDC) were added and incubated for 20 min. Modified beads were washed twice with 50 μL coupling solution (50 mM 2-(N-morpholino) ethanesulfonic acid (MES)) and resuspended in 20 μL of the same solution. Ten microliters of antibody solution (1 mg/mL) were added to the beads, and with coupling solution, the final volume was adjusted to 100 μL. Antibody coupling was done by incubating for 2 h with gentle rotation. After the coupling reaction, antibody-conjugated beads were washed twice with 200 μL of storage solution (Phosphate buffered saline (PBS) containing 0.05% Tween 20, 0.1% bovine serum albumin (BSA) and 0.05% sodium azide). The resulting antibody–bead conjugate in 100 μL of the solution was counted and stored at 2–8 °C (in the dark).

### 2.5. Multiplexed Microsphere Bead-Based Immunoassay

A bead-based sandwich immunoassay was developed as a 3-plex assay for NNMT, LCP1, and NM23A using bead-capture antibody conjugates, biotin-conjugated detection antibodies, and phycoerythrin-conjugated streptavidin (Invitrogen, MA, USA). The assay was performed in a 96-well assay plate (SPL Life Sciences, Billerica, MA, USA) at room temperature. For the entire assay procedure, incubations were done by agitating at 500 rpm. Throughout all assay steps, assays were done in an all-in-one reaction solution without a solution draining and washing step. Recombinant proteins and bovine serum were used to prepare standard calibrators and their dilution matrix as a mimic of the human serum samples. Bead suspensions containing 1000 conjugated beads in 40 μL of assay solution (50 mM 4-(2-hydroxyethyl)-1-piperazineethanesulfonic acid (HEPES)), 150 mM NaCl, 1% BSA, and 5 mM CaCl_2_) were transferred to each well of the assay plate and 10 μL of serum samples or calibration solutions were added and incubated for 20 min. Ten microliters of biotin-labeled antibody (0.02 mg/mL of anti-NNMT IgG, 0.04 mg/mL of anti-LCP1 IgG, 0.02 mg/mL of anti-NM23A IgG) was added and incubated for an additional 20 min. Without washing, 10 μL of phycoerythrin (PE)-conjugated streptavidin (20 μg/mL) in incubation solution was added and incubated for 25 min. Without washing, 70 μL of dilution solution containing 20mM HEPES pH7.5, 150 mm NaCl, 0.1% Tween 20, and 2.5% ethanol was added and mixed thoroughly for 5 min. The median fluorescence intensity (MFI) of reaction products was read with the Luminex^100^ system.

### 2.6. Analytical Performance Validation

The analytical performance of this 3-plex assay for NNMT, LCP1, and NM23A in human serum were assessed and validated under the guidelines from the International Medical Device Regulators Forum (http://www.imdrf.org). Assessment of the accuracy of measurement, the sensitivity of analysis, measuring range, and linearity of the assay were included in the validation of analytical performance. For assessment of trueness of accuracy, the analytes (triple markers) spiked in pooled normal human serum were assayed at low, middle, and high concentration, and recovery was calculated. The assessment of repeatability and reproducibility for the precision of accuracy was also performed. Repeatability assessment was done by measuring within-run variability with analytes in the calibration matrix and human serum. Variability was presented in terms of coefficient of variation (%CV). Reproducibility estimation was performed by measuring the variation of between-run assays on three independent days. For assessment of the analytical performance of sensitivity, the limit of quantification (LoQ) and limit of detection (LoD) were also calculated. LoD was measured by adding 3 standard deviations (SD) to the mean value of 11 samples of the blank matrix. Low LoQ (LLoQ) was estimated by measuring the mean value of 11 samples of whole range calibration solution. Acceptance criteria for LLoQ were relative error (RE) < ±20% and CV < 20%. For linearity of dilution, a serial dilution of calibrators was measured, and the slope was calculated. The dilution of calibrators was done with a matrix or with human serum in the series of seven 3-fold serial dilutions for the whole range of calibration solutions.

### 2.7. Data Analysis

For statistical analyses, MedCalc software (Ver.12.3.0.0, http://www.medcalc.be) was used. Pearson correlation coefficient (R^2^) was used for linear regression. For assessment of the significance of the difference in concentration of serum tumor markers between control individuals and RCC patients, the Mann–Whitney test (independent samples) was used as a rank-sum test. Receiver operating characteristic (ROC) analysis was used for diagnostic sensitivity, specificity, and accuracy (AUC) analysis. An algorithm score generation was adopted to facilitate the evaluation of the diagnostic performance of a three-marker combination. The scoring procedure for determining the best cut-point was described in a previous study [10]. In brief, the best cut-points for serum tumor marker concentrations were determined at the highest criteria of the Youden Index using ROC analysis of the training group (consisting of control and cancer sample donors). Based on the serum tumor marker concentration from the 3-plex assay, individuals were assigned a score of 0 ( ≤ cut-point) or 1 (> cut-point) for each marker, and as the sum of three markers, a score ranging from 0 to 3 was finally assigned. The best cut-point (cut-off) concentration of the three markers used in this study was 147 pg/mL for NNMT, 1780 pg/mL for LCP1, and 520 pg/mL for NM23A, respectively, as determined in a previous [10] and unpublished study.

## 3. Results

### 3.1. Validation of Analytical Performance

In our previous study, the analytical performance of this three-plex assay was assessed and validated with human plasma samples [10]. In this study, all the samples to be tested were human serum specimens. Then, validation of the analytical performance of this assay for NNMT, LCP1, and NM23A in human serum was performed and validated (Supplementary Materials S1).

For a within-run and between-run assay, acceptance criteria (%CV) were within 20%. The within-run assay for the precision of repeatability and the between-run assay for reproducibility through three independent days were within 3.4–19.8% and within 1.1–17.1%, respectively. The precision of repeatability of the assay with the matrix (3.6–19.8%) and human serum (3.4–17.6%) was comparable. The acceptance criteria (%CV) for trueness of accuracy were below ±10%. The concentration of three markers spiked in human normal serum was assayed and the recovery was calculated. The recovery of NNMT at low (296 pg/mL), middle (2667 pg/mL) and high (24,000 pg/mL) concentration was 108% (88–116%, 95% confidence interval), 92% (77–102%) and 102% (91–112%), respectively. At all three low, middle, and high concentrations, the mean recovery triple marker of the three-plex assay was within ±10%. Because of the analytical sensitivity from estimated LoD and LLoQ, the three-plex assay appeared to be highly sensitive. The LoD for NNMT, LCP1, and NM23A was 76.7 pg/mL, 193 pg/mL, and 147.5 pg/mL, respectively. Based on the acceptance criteria for precision (CV < 20%) and bias (RE < ±20%) of the three-plex assay, LLoQ was determined as the lowest value among each concentration of calibrators. The estimated LLoQ was 33 pg/mL (NNMT), 370 pg/mL (LCP1) and 99 pg/mL (NM23A). To assess the linearity of the dilution, each concentration of whole range calibrators from a three-fold serial dilution with human serum or matrix was measured. The slope representing the linearity of dilution for the three markers in human serum was over 0.991 and the slope of whole range of calibrators in the matrix of bovine serum was comparable to the slope of human serum (see Supplementary Materials S1) within the concentration range of 199–24,831 pg/mL (NNMT, R^2^ = 0.998), 10,783–105,263 pg/mL (LCP1, R^2^ = 0.991) and 16–23,784 pg/mL (NM23A, R^2^ = 0.998).

### 3.2. Differential Serum Levels of NNMT, LCP1 and NM23A in Control Individuals and RCC Patients

**Figure 1:**
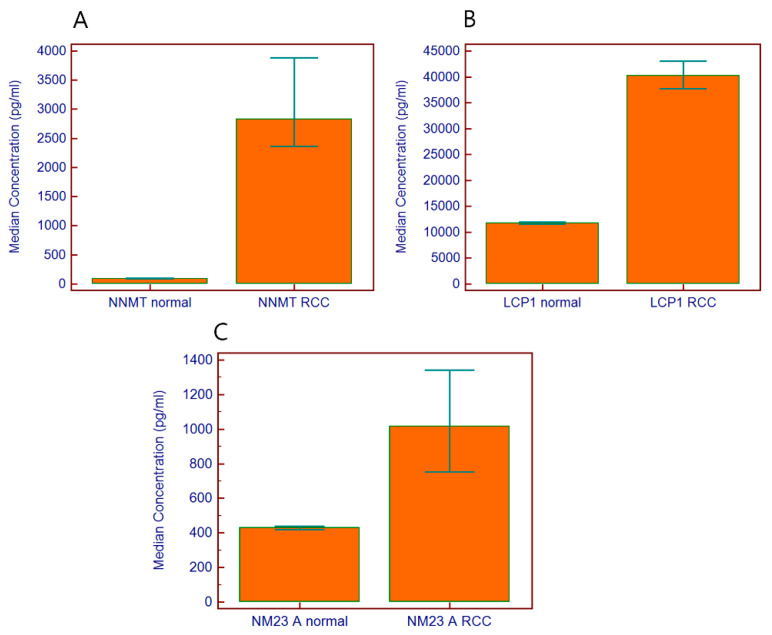
The serum concentration of NNMT, LCP1, and NM23A was measured with the 3-plex assay. The median concentration of NNMT was 30.1-fold higher in patients (2830 pg/mL) with RCC than in controls (94 pg/mL) (**A**); the median concentration of LCP1 was 3.4-fold higher in RCC patients (11,800 pg/mL in controls, 40,232 pg/mL in patients) (**B**); and the median concentration of NM23A was 2.4-fold higher in RCC patients (429 pg/mL in controls, 1016 pg/mL in patients) (**C**).

To evaluate their potential as tumor markers for RCC, serum concentrations of three tumor markers, NNMT, LCP1, and NM23A, were assayed with a total of 1042 specimens from 500 control healthy subjects and 542 patients with RCC of pathological stages I–IV. Serum levels of these tumor markers were increased in patients with RCC, as shown in Figure 1. The median concentration of NNMT was 2830 pg/mL in patients with RCC and 94 pg/mL in controls and appeared to be significantly increased in patients (*p* < 0.0001, Mann–Whitney test). Based on the assayed serum concentration of controls and patients, the diagnostic performance was assessed and its characteristics were represented by ROC analysis as shown in Table 2. For NNMT, the diagnostic sensitivity was 77.4% and AUC was 0.833 at a fixed specificity of 90%. The median concentration of LCP1 in controls and subjects with RCC was 11,800 pg/mL and 40,232 pg/mL, respectively. The median concentration of NM23A in controls and subjects was 429 pg/mL and 1016 pg/mL, respectively. The ratio of the concentration of LCP1 (3.4 fold) and NM23A (2.4 fold) between controls and subjects with RCC was lower than that of NNMT (30.1 fold), however the difference in concentration of LCP1 (*p* < 0.0001) and NM23A (*p* < 0.0001) was significant. At the specificity of 90%, the diagnostic sensitivity of LCP1 and NM23A was 67.3% and 41.3%, respectively.

### 3.3. Composite Markers (NNMT, LCP1 and NM23A Together) Showed Improved Clinical Performance

Using an algorithm for score generation, the diagnostic performance of the three-marker combination was evaluated. As described earlier, the cut-point was determined from plasma specimens composed of 189 plasma samples and samples of another training group from an unpublished study [10]. In this study, a previously determined cut-point was used for evaluation. The cut-points for NNMT, LCP1, and NM23A were 147 pg/mL, 17,800 pg/mL, and 520 pg/mL, respectively. The diagnostic performance of a combination of markers was evaluated with the summed score of the three markers. As described in the Material and Methods section, based on the serum tumor marker concentration of the three-plex assay, individuals were assigned a score of 0 (≤ cut-point) or 1 (> cut-point) for each marker, and a score ranging from 0 to 3 was finally assigned as the sum of the three markers. If the score was 2 or 3, it was determined as possibly “patient”, and if the score was 0 or 1, it was determined as possibly “healthy”. The resulting diagnostic characteristics for 1042 overall blind subjects are summarized in Table 2. As a single marker, NNMT and LCP1 showed better diagnostic performance than NM23A. The diagnostic accuracy (AUC) of NNMT and LCP1 was 0.833 and 0.844, respectively, and at a fixed specificity of 90%, the sensitivity of NNMT and LCP1 was 77.4% and 67.3%, respectively. As a composite three-marker, the diagnostic performance of the NNMT, LCP1, and NM23A combination was improved. At a defined specificity of 90%, the sensitivity of the three-marker assay was 87% and the diagnostic accuracy (AUC) was 0.917. The positive and negative predictive value of the three-marker assay was 87.2% (PPV) and 89.9% (NPV).

## 4. Discussion

As described earlier, several tumor marker candidates of RCC, including nicotinamide N-methyltransferase (NNMT), L-plastin (LCP1), and non-metastatic cells 1 protein (NM23A), were identified and verified. Also, a potential RCC tumor marker was selected and validated through evaluation with the developed plasma tumor marker assay. Among the candidate RCC tumor markers, NNMT, LCP1, and NM23A showed the highest features insignificance of difference in patients, the highest specificity in RCC compared to healthy individuals and several other cancers, and the best performances in the combination assay [9,10].

NNMT is a cytosolic enzyme and is mainly expressed in the human liver [12,13,14]. NNMT catalyzes xenobiotics and mediates the methylation reaction of nicotinamide and similar compounds using S-adenosyl methionine as the methyl donor to produce S-adenosyl-l-homocysteine and 1-methylnicotinamide [13,14]. N-methylation is a method by which drugs or other xenobiotics are metabolized by the liver [14]. NNMT expression in adipose tissue is associated with obesity and insulin resistance, and in embryonic stem cells, the expression of NNMT is believed to help maintain cells in a naive state [14].

An association between the elevated expression of NNMT and several cancers, including pancreatic and colorectal cancers, has been reported [15,16,17,18,19], and the potential of NNMT as a blood tumor marker for bladder and colorectal cancer has been suggested [17,20]. Furthermore, evidence was also provided that there is a correlation between the downregulation of NNMT in cells and lower rates of cell migration [15]. NNMT expression is upregulated in pancreatic cancer, where levels of the NNMT enzyme correlate with an increased risk of death. The cause of these correlations has not been established but may be related to the fact that the NNMT enzyme is an inhibitor of DNA repair [19]. Although elevated expression of NNMT has been reported in several cancers, NNMT expression was prominent in RCC. NNMT expression in solid tissue of RCC was especially high in comparison with several other cancer tissues, including cervical, lung, liver, and ovarian cancer [9]. Plastin is one of the actin-binding proteins. Among the L- and T-isoforms of plastins, LCP1 is the L-isoform of plastin that has been found in many malignant tumors, suggesting an association of its expression with tumorigenesis. In contrast, the T-isoform has been found in many normal cells [21,22,23]. The transcript level of NM23A is decreased in metastatic cells [24], and there are several studies on the association of NM23A with various cancers [24,25,26,27].

**Figure 2:**
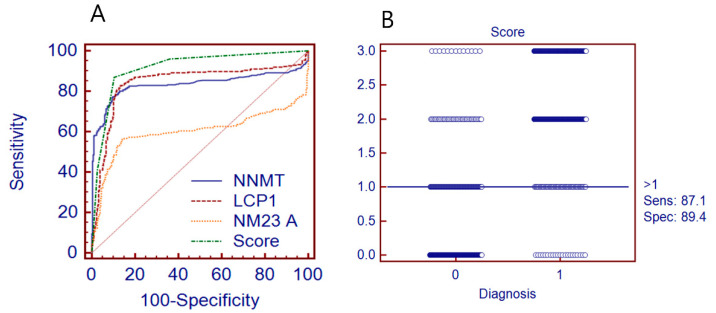
Comparison of Receiver Operating Characteristic (ROC) curve for NNMT, LCP1 and NM23A and the combination assay: (**A**) ROC curve for each of the three markers and scores from the composite assay and (**B**) dot plot of ROC for score from the composite assay. NNMT, Nicotinamide N-methyltransferase; LCP1, L-plastin; NM23A, Nonetastatic cells 1 protein; Sens, Sensitivity; Spec, Specificity.

The diagnostic characteristics of specificity and sensitivity for NNMT, LCP1, and NM23A or the three marker combination is shown in Figure 2A,B. There was an increased gain of ROC curve area through the varying specificity region, which led to an increased AUC value of the composite three-marker assay (0.917) compared with the diagnostic performance of each single marker alone (NNMT (0.833), LCP1 (0.844), and NM23A (0.601)). The sensitivity of the three-marker assay was high for most of the subtypes of RCC examined, except that the sensitivity of LCP1 for the papillary subtype case was the highest (Table 3). The sensitivity of the three-marker assay was in the range of 86% to 88% in detecting RCC regardless of subtype. When using serum samples as a biomarker specimen, there was no clear difference in analytical and clinical sensitivity in comparison with plasma samples. This result was consistent with a previous study [10] performed with a combined test group of 289 plasma samples from healthy donors and kidney cancer patients. In these two case studies, there was a consistent increase in sensitivity when these three markers were applied in a combination assay. In contrast to the diagnostic performance of composite or single-marker assays to overall subtypes of RCC, there was no specific discrimination of markers to certain types of subtypes of RCC.

**Figure 3:**
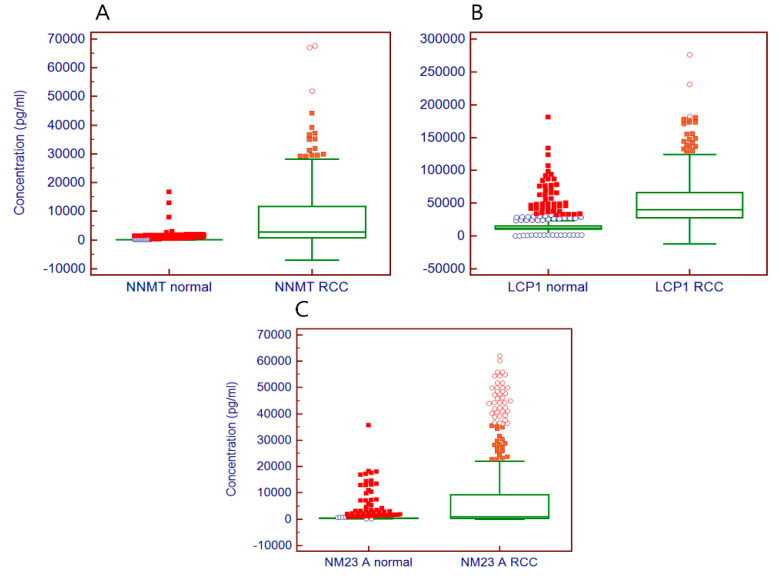
Distribution of serum concentration of NNMT (**A**), LCP1 (**B**), and NM23A (**C**) measured with the 3-plex assay. A box-and-whisker plot was used to represent the distribution of the three markers. The central box represents the values from the lower to upper quartile (25th to 75th percentile). The middle line represents the median. NNMT, Nicotinamide N-methyltransferase; LCP1, L-plastin; NM23A, Nonetastatic cells 1 protein.

In a previous study, we showed that NNMT, a cytosolic protein, could be detected in plasma, and its plasma level was significantly higher (6.2-fold) in subjects with RCC [10]. Now, in this present study, we confirmed that the serum concentration of three markers (NNMT, LCP1, and NM23A) greatly increased 30.1-fold, 3.4-fold, and 2.4-fold, respectively (Figure 3). The distribution of the serum concentration of NNMT is shown in Figure 3A. Although the differences in serum concentration of these tumor markers were significant, there was some overlap between serum levels of control individuals and patients with RCC. This could have originated from the abnormal expression of tumor markers associated with other unknown diseases without renal tumors, or from the analytical non-specificity of the assay. Regardless, there was a certain extent of non-specificity of these tumor markers and this is well-known as a limitation of the single tumor marker assay. A multi-marker assay might be a way to overcome these limitations of a single marker, as is the case in this study. This three-marker assay improved the diagnostic sensitivity and accuracy at a defined specificity (Table 2). Consequently, the limitations of a single-marker assay could be overcome, leading to improved diagnostic accuracy (0.917 AUC). In the present study, three markers and their combination assay discriminated early-stage kidney cancer with high sensitivity comparable to the late pathological stage of kidney cancer (Table 4). The serum concentration of the three markers was not correlated to the pathological stage. As shown in Table 1 and Table 5, there was a difference in the percentage of the population between control samples and patient samples, and also between sexes. This could cause the differences seen in the three marker profiles, including the resulting profiles of diagnostic characteristics. For this reason, the specificity of the control samples between males and females, and control samples according to the distribution by age, was examined (Table 5). The percentage of control samples between males and females was 38% and 62%, and the specificity was 91.7% and 88.1%, respectively. The specificity of males was somewhat higher than females. However, there was no relationship in the control samples according to age. In contrast, significant differences in sensitivity were not found in patient samples between males (88.6%) and females (88.0%). For the patient sample profiles, the averaged sensitivity of samples from younger patients (74–86% in 20–50s) differed from that in older patients (91–100% in 60–80s). Given the results in the present study, the lower percentage (38%) but higher specificity of male control samples could have contributed to the total sum of specificity (up to no more than 3.6% specificity). Although this does not appear to be the case in control samples, we thought that the probability of an ontogenetic effect of the three-marker assay on sensitivity in patient samples could not be entirely excluded. However, this remains to be unveiled with a more extensive population study.

## 5. Conclusions

From present scaled-up samples, multi-center study, as a potential serum/plasma tumor marker, NNMT, LCP1 and NM23A assay and its combination assay was validated to be efficient to detect early RCC. 

## Figures and Tables

**Table 1:** Participant demographics.

Combined Total (n = 1042)				Test Group (n = 1042)					
Control (n = 500)				Control (n = 500)					
Healthy (n = 500)				500 (100%)					
Benign (n = 0)				0 (0%)					
Sex	Male	Female	Age Median						
	188 (38%)	312 (62%)	46.8						
RCC (n = 542)				RCC (n = 542)					
Sex	Male	Female	Age Median						
	352 (65%)	190 (35%)	60						
Cell Type				Pathologic T(pT) Stage					
					I	II	III	IV	Missing
Clear Cell RCC (n = 351)				351 (64.8%)	268	39	40	3	1
Papillary RCC (n = 96)				96 (17.7%)	83	6	6	1	0
Chromophobe RCC (n = 71)				71 (13.1%)	55	10	6	0	0
Unclassified/Other RCC (n = 25)				25 (4.6%)	15	3	2	0	5
	Total				421(77.5%)	58(10.7%)	54(9.9%)	4(0.7%)	6(1.1%)

RCC, Renal Cell Carcinoma.

**Table 2:** Diagnostic characteristics of each of the three markers alone and a composite of the three markers of RCC.

Markers	NNMT	LCP1	NM23A	NNMT, LCP1, NM23A Score
Cut-point for scoring (pg/mL)	>147	>17,800	>520	>1
		Combined Total Group (n = 1042)		
Median Concentration (pg/mL) (range, lower and upper 95% CI)				
Control group (n = 500)	94.0 (89.6–99.0)	11,800.5 (11,566.11–11,984.2)	429.0 (419.5–439.0)	
Kidney cancer group (n = 542)	2830 (2361.3–3886.2)	40,232.5 (37,706.7–40,232.0)	1016.5 (753.1–1340.4)	
Mann–Whitney test	*p* < 0.0001	*p* < 0.0001	*p* < 0.0001	
AUC (95% CI))	0.833 (0.809–0.855)	0.844 (0.820–0.865)	0.601 (0.570–0.631)	0.917 (0.898–0.933)
Sensitivity (%) (95% CI)	90.2 (87.4–92.6)	82.5 (79.0–85.6)	56.2 (52.0–60.5)	87.1 (84.0–89.8)
Specificity (%) (95% CI)	77.1 (73.4–80.6)	86.8 (83.5–89.6)	85.8 (82.4–88.7)	89.4 (86.4–92.0)
+PV (%) (95% CI)	89.5 (86.4–92.1)	87.1 (83.9–89.9)	81.1 (76.8–84.9)	87.2 (80.0–92.5)
−PV (%) (95% CI)	78.4 (74.8–81.7)	82.0 (78.5–85.2)	64.4 (60.6–68.1)	89.9 (87.0–92.3)
Specificity = 90% Sensitivity (%) (95% CI)	77.4 (73.7–80.9)	67.3 (63.2–71.3)	41.3 (41.1–49.7)	87.0 (86.4–92.0)

RCC, Renal Cell Carcinoma; NNMT, Nicotinamide N-methyltransferase; LCP1, L-plastin; NM23A, Nonetastatic cells 1 protein; +PV, positive predictive value; −PV, negative predictive value; AUC, Area Under Curve.

**Table 3:** Sensitivity of three single markers and three-marker combination in subtypes of RCC.

	Sensitivity %		
	Cell Type		
Marker	Clear Cell Carcinoma	Papillary Carcinoma	Chromophobe Carcinoma
NNMT at cut-off > 147 pg/mL	81 (285/351)	84 (81/96)	85 (60/71)
LCP1 at cut-off > 17,800 pg/mL	78 (274/351)	89 (85/96)	83 (59/71)
NM23A at cut-off > 520 pg/mL	56 (196/351)	57 (55/96)	45 (32/71)
NNMT + LCP1 + NM23A score at cut-off > 1	86 (302/351)	88 (84/96)	86 (61/71)

RCC, Renal Cell Carcinoma; NNMT, Nicotinamide N-methyltransferase; LCP1, L-plastin; NM23A, Nonetastatic cells 1 protein.

**Table 4:** The specificity and sensitivity of NNMT or three-marker assay in several pathological tumor stages of Renal Cell Carcinoma (RCC).

	Specificity (%)	Sensitivity (%)			
	Control		Pathologic T(pT) stage		
		Stage I	Stage II	Stage III	Stage IV
NNMT at cut-off > 147 pg/mL	83.0 (415/500)	83 (312/375)	78 (64/82)	82 (32/39)	85 (33/39)
NNMT, LCP1, NM23A score at cut-off > 1	89.4 (447/500)	87 (326/375)	89 (73/82)	87 (34/39)	85 (33/39)

**Table 5:** Specificity or sensitivity according to age distribution and sex percentage for normal samples and patient samples.

Control Samples (n = 500)						Patient Samples (n = 542)						
Percentage of population by age						Percentage of population by age						
20s	30s	40s	50s	60s	70s	20s	30s	40s	50s	60s	70s	80s
15%	12%	28%	26%	17%	0.6%	2%	4%	10%	25%	38%	17%	3%
Specificity (%)						Sensitivity (%)						
92.3%	90.5%	86.5%	90.0%	89.7%	100%	75%	86%	74%	85%	92%	91%	100%
Percentage of population by sex				Specificity		Percentage of population by sex				Sensitivity		
Male	Female			Male	Female	Male	Female			Male	Female	
38%	62%			91.7%	88.1%	65%	35%			88.6%	88.0%	

## Supplementary Materials

The following are available online at https://www.mdpi.com/2075-4418/10/10/750/s1, S1. Supplement data.
