# Supplementary material for: Scale-Up Evaluation of a Composite Tumor Marker Assay for the Early Detection of Renal Cell Carcinoma
Source: Diagnostics (Basel). 2020 Sep 25;10(10):750. doi: 10.3390/diagnostics10100750 (PMC7601868; doi:10.3390/diagnostics10100750)
Supplement: Supplementary file 1 [file diagnostics-10-00750-s001.pdf]

S1. Supplement data.

S1. 1. Accuracy of measurement

Marker : NNMT

Spike Concentration(pg/ml)	Sample 1	Sample 2	Sample3	Sample4	Sample5	Sample6	Mean	SD	CV(%)
**24000**	25277	23684	26980	23966	21991	24571	24412	1525.5	6.2
**2667**	2777	2474	2414	2434	2736	2111	2491	221.9	8.9
**296**	378	418	357	378	378	317	371	30.1	8.1
**0**	55	75	35	41	43	55			
**Blank subtracted**									
**24000**	25222	23609	26946	23925	21948	24516			
**2667**	2722	2399	2379	2393	2694	2056			
**296**	323	343	323	337	335	262			
**Recovery(%)**									
**24000**	105	98	112	100	91	102	**102**		
**2667**	102	90	89	90	101	77	**92**		
**296**	109	116	109	114	113	88	**108**		

Marker : LCP1

Spike Concentration(pg/ml)	Sample1	Sample2	Sample3	Sample4	Sample5	Sample6	Mean	SD	CV(%)
**90000**	109400	105225	107925	115800	117200	119875	112571	5335.5	4.7
**10000**	25535	25058	26172	25814	25754	24571	25484	527.8	2.1
**1111**	16089	16687	15843	16547	16353	16101	16270	288.9	1.8
**0**	15388	15737	14816	15539	15523	14356			
**Blank subtracted**									
**90000**	94012	89488	93109	100261	101677	105519			
**10000**	10148	9321	11355	10275	10231	10215			
**1111**	702	950	1027	1008	831	1745			
**0**									
**90000**	104	99	103	111	113	117	**108**		
**10000**	101	93	114	103	102	102	**103**		
**1111**	63	86	92	91	75	157	**94**		

Marker : NM23A									
**Spike Concentration(pg/ml)**	Sample 1	Sample 2	Sample3	Sample4	Sample5	Sample6	Mean	SD	CV(%)
**24000**	25556	24993	27068	27068	25629	26569	25840	1054.0	4.1
**2667**	2643	2476	2478	2665	2372	2395	2528	118.6	4.7
**296**	404	409	380	399	393	404	384	36.7	9.6
**0**	97	129	45	92	39	132			
**Blank subtracted**									
**24000**	25458	24864	27024	26976	25590	26436			
**2667**	2546	2348	2433	2573	2333	2263			
**296**	307	280	335	307	354	272			
**Recovery(%)**									
**24000**	106	104	113	112	107	110	**109**		
**2667**	95	88	91	96	87	85	**91**		
**296**	104	95	113	104	120	92	**104**		

S1. 2. Within-run precision

Day1

Marker : NNMT						
	Calibrator Concentration in standard Matrix(pg/ml)			Calibrator Concentration in human serum(pg/ml)		
**Sample No.**	8000	889	99	8000	889	99
**1**	7901	1008	92	7942	770	100
**2**	8311	648	143	8307	822	128
**3**	9008	653	86	7775	881	72
**4**	8352	998	118	8580	844	114
**5**	8577	810	99	8778	998	92
**6**	9120	909	86	8504	1138	94
**Mean**	8545	838	104	8314	909	100
**SD**	418.9	147.5	20.5	353.9	124.2	17.6
**CV(%)**	4.9	17.6	19.7	4.3	13.7	17.6

Marker : LCP1

	Calibrator Concentration in standard Matrix(pg/ml)			Calibrator Concentration in human serum(pg/ml)		
**Sample No.**	8000	889	99	8000	889	99
**1**	30474	4646	442	46365	12545	11733
**2**	28697	3460	480	38465	12858	12795
**3**	23105	4066	429	41465	13070	12233
**4**	42184	3333	354	43340	14295	14158
**5**	39553	2980	303	44365	12545	13045
**6**	36658	2689	530	45865	14733	13345
**Mean**	33445	3529	423	43311	13341	12885
**SD**	6611.6	656.6	75.7	2703.5	858.4	776.6
**CV(%)**	19.8	18.6	17.9	6.2	6.4	6.0

Marker : NM23A

	**Calibrator Concentration** in standard Matrix(pg/ml)			**Calibrator Concentration** in human serum(pg/ml)		
**Sample No.**	8000	889	99	8000	889	99
**1**	7066	1032	110	8296	847	65
**2**	7814	840	136	8187	785	110
**3**	7699	782	74	8880	951	108
**4**	7595	718	101	8327	771	95
**5**	7026	861	101	9113	883	91
**6**	7427	988	92	8860	866	90
**Mean**	7438	870	102	8610	851	93
**SD**	300.9	109.5	18.7	352.9	60.7	14.8
**CV(%)**	4.0	12.6	18.3	4.1	7.1	15.9

Day 2

Marker : NNMT						
	Calibrator Concentration in standard Matrix(pg/ml)			Calibrator Concentration in human serum(pg/ml)		
**Sample No.**	8000	889	99	8000	889	99
**1**	8288	498	132	7972	1038	72
**2**	8528	542	108	8747	959	100
**3**	8659	593	116	7623	752	128
**4**	8004	582	152	7942	831	86
**5**	7655	652	116	7425	1078	94
**6**	8266	660	100	8246	966	100
**Mean**	8233	588	121	7993	937	97
**SD**	331.5	57.2	16.9	427.3	113.1	16.9
**CV(%)**	4.0	9.7	14.0	5.3	12.1	17.5

Marker : LCP1

	Calibrator Concentration in standard Matrix(pg/ml)			Calibrator Concentration in human serum(pg/ml)		
**Sample No.**	8000	889	99	8000	889	99
**1**	31189	2879	349	53390	13070	12658
**2**	31094	2904	340	48415	12920	13083
**3**	31094	2677	410	46090	13483	13583
**4**	27274	2702	440	48890	14183	11408
**5**	26849	2664	286	43465	17970	13458
**6**	25151	3662	386	43890	13345	13733
**Mean**	28775	2915	368	47357	14162	12987
**SD**	2438.6	347.3	50.0	3382.3	1749.5	790.0
**CV(%)**	8.5	11.9	13.6	7.1	12.4	6.1

Marker : NM23A

	Calibrator Concentration in standard Matrix(pg/ml)			Calibrator Concentration in human serum(pg/ml)		
**Sample No.**	8000	889	99	8000	889	99
**1**	8270	664	98	7587	951	92
**2**	7984	826	97	8023	836	79
**3**	7420	627	69	8525	766	86
**4**	7670	782	93	10176	868	81
**5**	7860	900	87	8525	908	109
**6**	8142	961	107	7532	881	95
**Mean**	7891	793	92	8395	868	90
**SD**	285.0	119.3	11.8	889.1	58.0	10.1
**CV(%)**	3.6	15.0	12.8	10.6	6.7	11.2

Day 3

Marker : NNMT						
	Calibrator Concentration in standard Matrix(pg/ml)			Calibrator Concentration in human serum(pg/ml)		
**Sample No.**	8000	889	99	8000	889	99
**1**	9041	700	109	8443	784	89
**2**	7414	839	70	7775	1070	61
**3**	7840	918	91	7988	839	86
**4**	8069	958	100	7562	998	103
**5**	7240	1050	91	7608	1149	72
**6**	7021	878	109	7957	831	100
**Mean**	7771	890	95	7889	945	85
**SD**	668.1	107.7	13.3	294.7	135.6	14.8
**CV(%)**	8.6	12.1	14.0	3.7	14.3	17.3

Marker : LCP1

	Calibrator Concentration in standard Matrix(pg/ml)			Calibrator Concentration in human serum(pg/ml)		
**Sample No.**	8000	889	99	8000	889	99
**1**	31377	3409	337	46215	13045	11420
**2**	21755	3813	325	48465	14045	11970
**3**	31660	3737	398	48865	13608	10358
**4**	22509	2652	380	48640	13220	13458
**5**	25717	2803	271	51090	14283	12920
**6**	34585	3384	380	46465	12933	13483
**Mean**	27934	3300	348	48290	13522	12268
**SD**	4873.4	436.1	42.8	1631.0	504.2	1138.2
**CV(%)**	17.4	13.2	12.3	3.4	3.7	9.3

Marker : NM23A						
	Calibrator Concentration in standard Matrix(pg/ml)			Calibrator Concentration in human serum(pg/ml)		
**Sample No.**	8000	889	99	8000	889	99
**1**	8005	692	96	7536	870	108
**2**	7429	838	99	8525	887	79
**3**	8098	652	84	10122	847	70
**4**	6986	810	102	9289	968	110
**5**	7473	887	64	7583	806	95
**6**	7580	981	107	7579	919	79
**Mean**	7595	810	92	8439	883	90
**SD**	373.0	111.6	14.3	987.3	51.8	15.2
**CV(%)**	4.9	13.8	15.5	11.7	5.9	16.8

S1. 3. Between-run precision

Marker : NNMT						
	Calibrator Concentration in standard Matrix(pg/ml)			Calibrator Concentration in human serum(pg/ml)		
**Sample No.**	8000	889	99	8000	889	99
**Day1**	8545	838	104	8314	909	100
**Day2**	8233	588	121	7993	937	97
**Day3**	7771	890	95	7889	945	85
**Mean**	8183	772	106	8065	930	94
**SD**	318	132	11	181	16	6
**CV(%)**	3.9	17.1	10.0	2.2	1.7	6.8

Marker : LCP1

	Calibrator Concentration in standard Matrix(pg/ml)			Calibrator Concentration in human serum(pg/ml)		
**Sample No.**	8000	889	99	8000	889	99
**Day1**	33445	3529	423	43311	13341	12885
**Day2**	28775	2915	368	47357	14162	12987
**Day3**	27934	3300	348	48290	13522	12268
**Mean**	30051	3248	380	46319	13675	12713
**SD**	2424	254	32	2161	352	318
**CV(%)**	8.1	7.8	8.3	4.7	2.6	2.5

Marker : NM23A

	Calibrator Concentration in standard Matrix(pg/ml)			Calibrator Concentration in human serum(pg/ml)		
**Sample No.**	8000	889	99	8000	889	99
**Day1**	7438	870	102	8610	851	93
**Day2**	7891	793	92	8395	868	90
**Day3**	7595	810	92	8439	883	90
**Mean**	7641	825	95	8481	867	91
**SD**	188	33	5	93	13	1
**CV(%)**	2.5	4.0	5.0	1.1	1.5	1.5

S1. 4. Linearity and Linearity of serial dilution

Marker : NNMT

				Mean					Mean
std	matrix	matrix	matrix	matrix	std	h spike	h spike	h spike	human spike
24000	21456	24212	24009	23226	24000	24849	24831	24813	24831
8000	6316	7577	7532	7142	8000	8443	7775	7988	8069
2667	1589	1741	2380	1903	2667	3580	3611	3595	3595
889	761	678	1133	857	889	784	1070	839	898
296	404	495	434	444	296	571	556	586	571
99	176	146	150	157	99	798	268	798	621
33	39	32	32	34	33	404	419	412	412
0	54	-12	-3	13	0	206	191	199	199

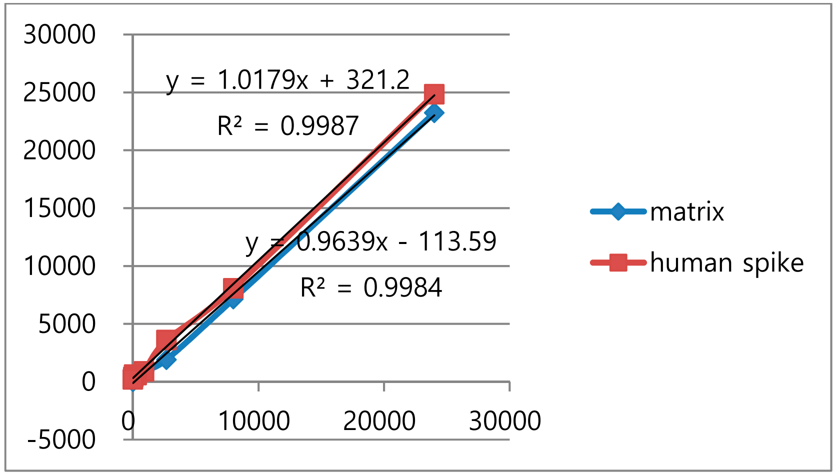

Marker : LCP1

				**Mean**					**mean**
Std	matrix	matrix	matrix	matrix	Std	h spike	h spike	h spike	human spike
90000	81896	86849	88736	85827	90000	105230	105180	105380	105263
30000	40245	33075	28453	33925	30000	46215	48465	48865	47848
10000	13925	14962	13358	14082	10000	28415	28390	28440	28415
3333	2874	2697	3152	2907	3333	13045	14045	13608	13566
1111	1005	904	803	904	1111	11670	11658	11683	11670
370	323	386	374	361	370	11420	11970	10358	11249
123	172	197	475	281	123	10445	10433	10458	10445
0	121	146	96	121	0	10795	10783	10770	10783

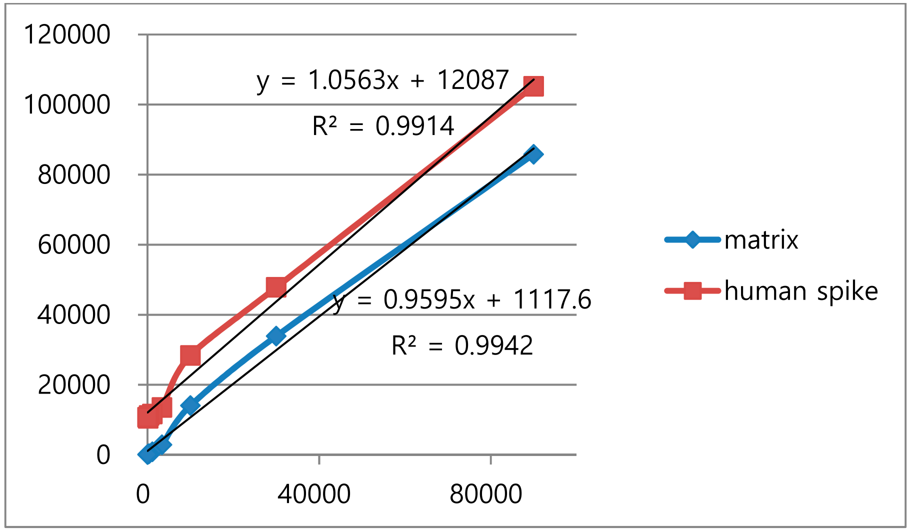

Marker : NM23A

				**Mean**					**Mean**
Std	matrix	matrix	matrix	matrix	Std	h spike	h spike	h spike	human spike
24000	22476	21867	20324	21556	24000	23803	23738	23811	23784
8000	7735	7817	7558	7703	8000	7536	8525	10122	8728
2667	3916	3554	3605	3692	2667	3214	3218	3210	3214
889	1124	982	1016	1041	889	870	887	847	868
296	318	340	260	306	296	222	219	218	220
99	110	112	92	104	99	108	79	70	86
33	61	70	163	98	33	29	27	25	27
0	167	25	114	102	0	16	18	14	16

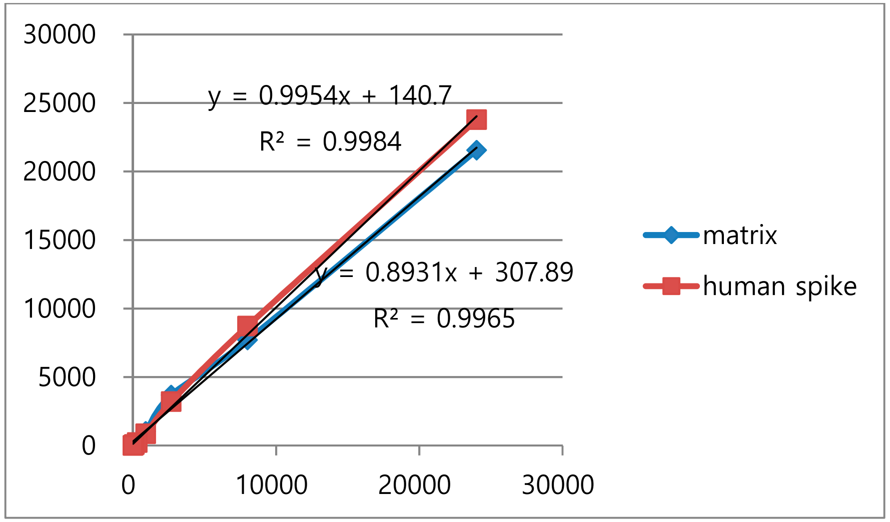

S1. 5. Limit of Detection(LoD) and Low Limit of Quantification(LLoQ)

Marker : NNMT

	S1	S2	S3	S4	S5	S6	S7	S8	S9	S10	S11	Mean	SD	CV(%)	RE(%)
**24000**	21456	24212	24009	22124	24463	19928	23651	24332	22565	22804	24320	23079	1392.0	6.0	3.8
**8000**	6316	7577	7532	5753	6939	7030	8216	7471	5890	8839	7091	7150	890.0	12.4	10.6
**2667**	1589	1741	2380	2760	2106	2197	2129	2273	2577	2098	2623	2225	340.1	15.3	16.6
**889**	761	678	1133	799	829	829	997	905	769	791	776	843	120.9	14.4	5.2
**296**	313	298	282	298	252	282	328	343	328	267	374	306	33.9	11.1	-3.2
**99**	100	115	115	100	130	115	115	100	130	146	115	117	13.7	11.7	-18.0
**33**	39	32	32	47	39	24	38	36	39	24	39	35	6.6	18.7	-7.1
**0**	54	-12	-3	-22	9	24	-14	6	-11	39	18	8	22.9		
					LoD(3 x SD + mead) = 3 x 22.9 + 8 =76.7 pg/ml										
					LLoQ(Lowest concentration < 20%(CV(%) = 33pg/ml										

Marker : LCP1

Std	S1	S2	S3	S4	S5	S6	S7	S8	S9	S10	S11	Mean	SD	CV(%)	RE(%)
**90000**	81896	86849	88736	76802	85104	79066	93500	88972	92132	95340	80717	86283	5842.4	6.8	4.1
**30000**	33170	33075	28453	39113	37038	33642	35717	39679	39066	39113	32887	35541	3437.0	9.7	-18.5
**10000**	13925	10245	13358	7038	13358	10434	8642	11755	14113	9868	12085	11347	2199.6	19.4	-13.5
**3333**	2874	2697	3152	4439	3455	3657	2949	3682	2773	4439	2912	3366	600.3	17.8	-1.0
**1111**	1005	904	803	955	1283	1030	980	1131	854	854	1157	996	140.1	14.1	10.4
**370**	323	386	374	348	424	500	525	399	475	374	525	423	68.6	16.2	-14.2
**123**	172	197	475	172	348	1030	803	71	273	-5	475	365	300.6	82.5	-195.3
**0**	121	146	96	121	71	96	71	96	71	121	20	94	33.1		
					LoD(3 x SD + mead) = 3 x 33.1 + 94 =193 pg/ml										
					LLoQ(Lowest concentration < 20%(CV(%) = 370pg/ml										

Marker : NM23A

Std	S1	S2	S3	S4	S5	S6	S7	S8	S9	S10	S11	Mean	SD	CV(%)	RE(%)
**24000**	24419	23757	22081	25248	23209	23589	25702	24777	23394	24678	24215	24097	978.9	4.1	-0.4
**8000**	7735	7817	7558	7606	8262	6822	6811	7943	8056	7801	7985	7672	446.2	5.8	4.1
**2667**	3207	3189	2644	2666	2613	3807	2458	3202	3304	3714	3393	3109	434.7	14.0	-16.6
**889**	1124	982	1016	699	1102	765	1137	783	814	748	1106	934	165.2	17.7	-5.1
**296**	318	340	260	318	211	234	251	216	300	225	260	267	43.2	16.2	10.0
**99**	110	112	92	105	74	87	87	87	105	65	70	90	15.4	17.0	8.4
**33**	61	70	163	43	48	-28	74	-6	-6	70	65	50	49.3	97.8	-53.0
**0**	56	65	114	39	21	-32	-14	-14	-14	21	8	23	41.5		
					LoD(3 x SD + mead) = 3 x 41.5 + 23 =147.5 pg/ml										
					LLoQ(Lowest concentration < 20%(CV(%) = 99pg/ml										
